# Cognitive-behavioral rehabilitation in patients with cardiovascular diseases: a randomized controlled trial (CBR-CARDIO, DRKS00029295)

**DOI:** 10.1186/s12872-023-03272-1

**Published:** 2023-05-15

**Authors:** Matthias Bethge, Friederike Thome-Soós, Luka Marko Rašo, Lisa Weier, Dieter Benninghoven

**Affiliations:** 1 Institute for Social Medicine and Epidemiology, University of Lübeck, Ratzeburger Allee 160, 23562 Lübeck, Germany; 2 Mühlenbergklinik Holsteinische Schweiz, Frahmsallee 1-7, 23714 Bad Malente, Germany

**Keywords:** Cardiac rehabilitation, Cardiovascular diseases, Psychosocial intervention, Behavioral symptoms, Emotions

## Abstract

**Background:**

Depression, generalized and cardiac anxiety, and posttraumatic stress disorder negatively affect disease severity, participation, and mortality in patients with cardiovascular disease. Psychological treatments within cardiac rehabilitation may improve the outcomes of these patients. We therefore developed a cognitive-behavioral rehabilitation program for patients with cardiovascular disease and mild or moderate mental illness or stress or exhaustion. In Germany, similar programs are well established in musculoskeletal rehabilitation and cancer rehabilitation. However, no randomized controlled trials have evaluated if such programs achieve better outcomes in patients with cardiovascular disease compared with standard cardiac rehabilitation.

**Methods:**

Our randomized controlled trial compares cognitive-behavioral cardiac rehabilitation with standard cardiac rehabilitation. The cognitive-behavioral program complements standard cardiac rehabilitation with additional psychological and exercise interventions. Both rehabilitation programs last for four weeks. We enroll 410 patients with cardiovascular disease and mild or moderate mental illness or stress or exhaustion aged 18 to 65 years. Half of the individuals are randomly assigned to cognitive-behavioral rehabilitation and the other half to standard cardiac rehabilitation. Our primary outcome is cardiac anxiety 12 months after the end of rehabilitation. Cardiac anxiety is assessed with the German 17-item version of the Cardiac Anxiety Questionnaire. Secondary outcomes cover outcomes assessed by clinical examinations and medical assessments and a range of patient-reported outcome measures.

**Discussion:**

This randomized controlled trial is designed to determine the effectiveness of cognitive-behavioral rehabilitation at decreasing cardiac anxiety in patients with cardiovascular disease and mild or moderate mental illness or stress or exhaustion.

**Trial registration:**

German Clinical Trials Register (DRKS00029295, June 21, 2022).

**Supplementary Information:**

The online version contains supplementary material available at 10.1186/s12872-023-03272-1.

## Background

Cardiovascular diseases are the most frequent cause of death and a major risk factor for health-related impairments of activities and participation [1]. About one in three people with a cardiovascular disease has at least one mental disorder in addition to the cardiovascular disease [2]. Persistent psychological impairment in particular increases the risk of mortality of patients with stable coronary heart disease [3, 4], and depression is associated with the degree of disability experienced by long-term survivors of myocardial infarction [5]. Early identification and treatment of psychological impairments is therefore an important objective of cardiac rehabilitation [6–8].

In Germany, cognitive-behavioral rehabilitation was developed for patients coping with a somatic illness who are also affected by psychological stress or comorbid psychological disorders. Cognitive-behavioral rehabilitation is a multimodal intervention that includes additional psychological and exercise interventions as compared to standard rehabilitation programs [9]. In patients with musculoskeletal disorders, randomized controlled trials revealed fewer depressive symptoms in the short and medium term among participants in cognitive-behavioral rehabilitation programs [10, 11] and reported better pain management strategies, lower pain intensity, and better general health following cognitive-behavioral compared with standard rehabilitation programs in the long term [10, 12]. In addition, a propensity-score-matched analysis showed slight benefits of cognitive-behavioral rehabilitation programs implemented in real-world care for patients with musculoskeletal disorders: Participants in cognitive-behavioral rehabilitation reported better self-rated work ability, better physical functioning, better self-management skills, lower pain impairment, and lower fear-avoidance beliefs 10 months after completing the program than comparable individuals receiving standard rehabilitation [13].

In a recent study, we tested the implementation of a cognitive-behavioral rehabilitation program for patients with cardiovascular disease [14]. Study participants were treated in the newly implemented cognitive-behavioral cardiac rehabilitation program or standard cardiac rehabilitation. The additional psychological group intervention was based on acceptance and commitment therapy. Those treated in the cognitive-behavioral program had stronger baseline mental health impairments than participants in the standard cardiac rehabilitation program. In addition, mental disorders were documented more frequently at baseline (especially neurotic disorders, stress disorders, and somatoform disorders). The fidelity of implementation was confirmed. Individuals treated in the cognitive-behavioral program also acknowledged more cognitive-behavioral elements, stronger consistency of the cognitive-behavioral approach, and more gains in competences to cope with the disease. The change in several mental and physical outcomes was substantial and corresponded to large standardized differences between the start and end of rehabilitation. To clarify whether cognitive-behavioral rehabilitation achieves better outcomes than standard cardiac treatment, we consider a randomized controlled trial necessary.

### Objectives

Our randomized controlled trial tests whether participants in the cognitive-behavioral rehabilitation program have more favorable outcomes, in particular mental health outcomes, than participants in standard cardiac treatment. Our primary outcome is cardiac anxiety measured with the Cardiac Anxiety Questionnaire 12 months after the end of rehabilitation [15, 16].

### Trial design

We are conducting a single-center, randomized controlled trial comparing two groups of cardiac rehabilitation patients. The intervention group receives a cognitive-behavioral rehabilitation program. The control group receives the standard cardiac rehabilitation treatment provided at the study center. Participants are randomly assigned to cognitive-behavioral rehabilitation or standard cardiac rehabilitation after giving informed consent.

The study has been registered on the German Clinical Trials Register (DRKS00029295). The items from the World Health Organization Trial Registration Data Set are available as Additional file 1. This paper contains the original study protocol. Any substantial modifications to the study protocol will be submitted to the Ethics Committee of the University of Lübeck for approval prior to implementation. These amendments will be documented in detail in the German Clinical Trials Register and described transparently in trial reports.

The trial is investigator initiated, and the University of Lübeck is the primary sponsor of the trial. An advisory board incorporating researchers, physicians, and employees of Federal German Pension Insurance and the German Pension Insurance North accompanies the conduct of the study and meets once a year.

## Methods

### Study setting

In Germany, rehabilitation due to cardiovascular diseases for people of working age is mainly provided by the pension insurance agencies and is intended to prevent health-related exit from work and disability pensions. Medical rehabilitation is possible both after acute events (e.g., post-acute rehabilitation after an acute myocardial infarction) and due to chronic diseases (e.g., chronic heart failure). There are 115 cardiac rehabilitation departments used by the pension insurance agencies [17], and in 2020, nearly 70,000 cardiac rehabilitations [17] – almost three-quarters of which were post-acute rehabilitations – were completed for people of working age. The utilization of cardiac rehabilitation is almost three times higher among men than women. The average age of patients with cardiovascular diseases whose rehabilitation is covered by the pension insurance agencies is about 55 years. The content of multi-professional cardiac rehabilitation includes exercise therapy (especially aerobic endurance training and strength training), patient and health education, nutrition therapy, psychological and relaxation interventions, and tobacco cessation. In order to ensure uniform and consistent minimum standards throughout Germany, therapy standards for rehabilitation of coronary heart disease have been formulated [18] and are based on the recommendations of the National Guideline for Coronary Heart Disease [19]. The minimum dose specified in the therapy standards for 12 treatment components corresponds to 29 to 34 treatment hours during a three- to four-week rehabilitation program [18]. The rehabilitation center where we recruit patients (https://www.muehlenbergklinik-holsteinische-schweiz.de) is located in northern Germany. It currently has 321 patient treatment slots, 130 of which are for cardiac rehabilitation. Thirty of the 130 treatment slots for patients with cardiovascular diseases are intended for cognitive-behavioral cardiac rehabilitation.

### Eligibility criteria

We are including patients aged 18 to 65 years who receive rehabilitation at our study center due to cardiovascular disease (ICD-10 I05 to I71 as well as I95 and I97) assigned by the Federal German Pension Insurance or the German Pension Insurance North and for whom mild or moderate mental illness or stress or exhaustion are recorded in the application documents for cardiac rehabilitation. For patients assigned by the Federal German Pension Insurance, mental illness or stress or exhaustion is determined by the socio-medical service of the Federal German Pension Insurance. For patients assigned by the German Pension Insurance North, this is determined by a cardiologist at the study center. The determination of mild or moderate mental illness or stress or exhaustion is performed in advance of the program with only the documents used to claim the rehabilitation.

Patients with severe mental illness (schizophrenia, schizoaffective disorder, bipolar disorder, mania, severe unipolar depression), severe heart failure (at least stage III according to the classification of the New York Heart Association), and significant limitations with the German language are excluded.

### Interventions

#### Cognitive-behavioral rehabilitation

The cognitive-behavioral rehabilitation program was developed for cardiac rehabilitation patients with mild or moderate mental illness or stress or exhaustion. The program complements standard cardiac rehabilitation with additional psychological and exercise interventions and follows the framework of cognitive-behavioral rehabilitation formulated by the German Pension Insurance [20].

Cognitive-behavioral rehabilitation is conducted as a group program, i.e., 8–12 patients who begin the rehabilitation program at the same time complete five specific additional cognitive-behavioral components together. Table 1 summarizes these five components, e.g., a psychological group, individual psychological sessions, relaxation training, a seminar on heart and psyche, and an exercise group. Moreover, Additional file 2 provides a detailed description of the five components according to the Template for Intervention Description and Replication [21]. The treatments beyond these five components follow – as in the control group – the recommendations for the rehabilitation of coronary heart disease published by the German Pension Insurance [18]. Cognitive-behavioral rehabilitation lasts for four weeks.

A detailed description of the psychological group can be found in Benninghoven et al. [14]. This group includes seven 90-minute sessions and is based on acceptance and commitment therapy [14, 22, 23]. It combines cognitive-behavioral therapy techniques with mindfulness- and acceptance-based methods and emotion-regulating strategies. In addition to complementary psychological and educational interventions, cognitive-behavioral rehabilitation is characterized by intensified exercise therapy, as there is strong evidence that exercise therapy reduces cardiovascular mortality and hospitalizations and improves quality of life [24].

**Table 1:** Components of the cognitive-behavioral rehabilitation program

Brief name	Psychological group	Individual psychological sessions	Relaxation training	Seminar: Heart and psyche	Exercise group
What (procedures)	The seven sessions cover, amongst others, mindfulness exercises, the ACT matrix, acceptance of negative thoughts, mental flexibility, personal values, and the elaboration of concrete plans for the time after rehabilitation. A more detailed overview of the seven sessions is presented in Additional File 2.	During the admission interview, anamnestic information (including contextual factors such as family, work, and biography) as well as the experience of illness and illness behavior are recorded, and the expectations of the rehabilitation patients are clarified. In addition, at the end of rehabilitation, mental disorders are assessed with a standardized short interview in order to specify further treatment recommendations.	Participants learn progressive muscle relaxation and develop individual ways to apply the technique in everyday life.	The following topics will be covered in the two sessions. Session 1: Structure and function of the heart and cardiovascular system; function of the autonomic nervous system and the hormonal system. Session 2: Control function of the brain and influence of the emotional state; influence of lifestyle and attitude changes on physical processes, especially on the cardiovascular system.	The exercise intervention includes an individual admission assessment by a physical therapist, cardiovascular exercise therapy, ergometer training, strength training, and coordination training in a group.
Who provided	A psychological psychotherapist or graduated psychologist in psychotherapy training leads the group throughout the rehabilitation and is the primary psychological contact. The primary physical therapist is present at the first and last group meetings.	Psychological psychotherapist or graduated psychologist in psychotherapy training	Psychological psychotherapist or graduated psychologist in psychotherapy training	Physician	The primary physiotherapist leads the group exercise sessions throughout rehabilitation.
How	Group intervention	Individual intervention	Group intervention	Group intervention	Group intervention
Where	Quiet group room	Workroom of the primary psychologist	Quiet group room/gym	Quiet group room in the rehabilitation facility	Gym
When and how much	During the rehabilitation program, seven sessions of 90 min each, in total 630 min	During the rehabilitation program, at least one 60-minute session	During the rehabilitation program, one session of 60 min and eight sessions of 30 min each, in total 300 min	During the rehabilitation program, two sessions of 60 min each, in total 120 min	During the rehabilitation program, individual admission assessment: one sessions of 30 min, cardiovascular exercise therapy: 20 sessions of 30 min each, ergometer training: one session of 60 min and 11 sessions of 30 min each, strength training: 6 sessions of 60 min each, coordination training: 10 sessions of 30 min each, in total 1680 min

#### Standard cardiac rehabilitation

Controls receive the standard cardiac rehabilitation program in accordance with the recommendations for the rehabilitation of coronary heart disease [18]. To ensure that any potential benefits of the cognitive-behavioral rehabilitation program are not due to a longer rehabilitation duration of the cognitive-behavioral program, the standard rehabilitation program is also conducted for four weeks instead of the usual three weeks.

### Outcomes

#### Primary outcome

Our primary outcome is cardiac anxiety 12 months after the end of rehabilitation. Cardiac anxiety is assessed with the German 17-item version of the Cardiac Anxiety Questionnaire [15, 16]. All items are rated from 0 to 4 points (“never” to “always”). Higher values represent more pronounced fear. The total score is calculated as the mean of eight items on fear, four items on avoidance, and five items on attention.

#### Secondary outcomes

##### Mental health

The three subscales of the Cardiac Anxiety Questionnaire, i.e., fear, avoidance, and attention, are collected as secondary outcomes [15, 16]. Furthermore, the level of change in cardiac anxiety and the reduction of cardiac anxiety by at least 0.2 points (5% of the scale range) is assessed. Other indicators of mental health include depression (0 to 27 points), generalized anxiety (0 to 21 points) and somatization (0 to 30 points). These outcomes are assessed using the Patient Health Questionnaire [25].

*Physical health*: Functional capacity is assessed with eight items of the IRES-24 (Indicators of rehabilitation status, German: Indikatoren des Reha-Status). The items assess difficulties in engaging in physical activities, e.g., sports that really make you sweat, such as jogging, skiing, and mountain hiking [26]. Difficulties are assessed on a 5-point scale (“impossible” to “no difficulty”). The total score ranges from 0 to 10 points. Higher values of the total score represent better functional capacity. Additionally, the rehabilitation center records systolic and diastolic blood pressure and ergometer performance in watts per kg at the beginning and end of rehabilitation.

*General health*: To describe general health, we use a visual analog scale with scores ranging from 0 to 100 points [27]. Furthermore, we use the 5-level EQ-5D, a preference-based measure of health-related quality of life, to assess general health [28, 29]. Using the 5-level EQ-5D, participants are asked to indicate their health status by answering questions from five different dimensions: mobility, self-care, usual activities, pain/discomfort, and anxiety/depression. Each dimension has five levels of severity: no problems, mild problems, moderate problems, severe problems, and extreme problems. Each number combination represents a health state, with 11111 reflecting no problems in any of the five dimensions. The 5-digit health status is then converted into an index value reflecting how good or bad an individual’s health status is, according to the preferences of the German general population [30]. The health state of 11111 corresponds to a value of 1.

##### Health behavior

Motivation to change lifestyle is assessed using a 5-point scale ranging from 1 (“certainly”) to 5 (“certainly not”), and we count who is motivated to change lifestyle (certainly or rather yes) [31]. Confidence about regular physical activity is evaluated using the Exercise Self-Efficacy Scale comprising 10 statements (e.g., “I am confident that I can accomplish physical activity and exercise goals that I set”). These statements are rated on a 4-point scale (“not at all true” to “always true”). A total score is calculated ranging from 10 to 40 points [32]. Higher scores represent a stronger belief to engage in regular physical activity. Smoking status is determined by three categories (“yes, I smoke”, “no, but I have smoked”, and “no, I have never smoked”) at baseline and by two categories at the end of the rehabilitation and at the 3-month and 12-month follow-up (“yes” or “no”). Body weight and height are aggregated to the body mass index in kg/m². Weekly physical activity is assessed for a maximum of three activities [33]. Participants state which physical activities were performed in the last four weeks. In addition, they provide the frequency and duration in minutes for each activity. Frequency and duration are multiplied for each activity, the dose of all activities is added up, and the sum is finally divided by four to calculate the weekly amount of physical activity.

##### Work ability and job situation

Self-rated work ability is measured using the Work Ability Score [34]. The Work Ability Score is the first item of the Work Ability Index and compares the current work ability with the lifetime best. The scale ranges from 0 („completely unable to work“) to 10 („work ability at its best”). The Work Ability Score correlates strongly with the total Work Ability Index score [35]. We also assess employment, sick leave status and cumulative sickness absence in the last 3 or 12 months (weeks). At the 3-month and 12-month follow-up, we ask when the participants were first working for at least four weeks without absenteeism (time to sustainable return-to-work) [36], and we assess if patients receive a disability pension. The physician’s assessment of work capacity for the last job and the general labor market at the end of rehabilitation are extracted from the standardized medical discharge reports [37].

##### Dose delivered

The dose delivered describes the number of intended intervention units provided to participants [38]. Treatments during the rehabilitation program are extracted from the participants’ standardized medical discharge reports, which document all treatments performed during rehabilitation using the classification of therapeutic services [39].

##### Dose received

The dose received describes the elements of the intervention the participants actually received [38]. We record the dose of the cognitive-behavioral rehabilitation received using three scores, which we used in a previous study evaluating the implementation of the approach [14]. These patient-reported experience measures assess the content of the cognitive-behavioral rehabilitation program (12 items, 0 to 12 points), the consistency of the approach (4 items, 0 to 16 points), and the gain in competence experienced by the participants (10 items, 0 to 40 points).

##### Health-related costs

For our health economic evaluation, we use the Questionnaire for Health-Related Resource Use in an Elderly Population [40]. The questionnaire assesses, among others, the type of health insurance, medications taken in the last seven days, outpatient physician visits and therapeutic services used in the last 3 months, as well as rehabilitation measures, outpatient surgery and treatments in outpatient or inpatient hospitals in the last 12 months. The health-related costs are calculated using the rates proposed by Bock et al. [41].

##### Recommendations for subsequent services

Recommendations for 12 common subsequent services are extracted from the standardized medical discharge report at the end of the rehabilitation [37].

##### Diagnoses

Diagnoses are also extracted from the standardized medical discharge report at the end of the rehabilitation [37]. In the intervention groups mental disorders are assessed using a structured clinical interview [42].

##### Sociodemographic data

These include data on native language, partnership, number of children, level of school education and vocational qualification.

Table 2 summarizes all outcomes, measurement instruments and the timing of the assessments.

**Table 2:** Measures, assessment, expected scaling, and measurement occasions

*Outcome*	*Source and reference*	*Scaling*	*Time*
**Primary outcome**			
Cardiac anxiety	Total score of the German 17-item version of the CAQ [16]	Continuously, 0 to 4 points	T0, T1, T2, T3
**Secondary outcomes**			
Fear	Subscale of the German 17-item version of the CAQ [16]	Continuously, 0 to 4 points	T0, T1, T2, T3
Avoidance	Subscale of the German 17-item version of the CAQ [16]	Continuously, 0 to 4 points	T0, T1, T2, T3
Attention	Subscale of the German 17-item version of the CAQ [16]	Continuously, 0 to 4 points	T0, T1, T2, T3
Change in score for cardiac anxiety	Total score of the German 17-item version of the CAQ [16]	Continuously, − 4 to 4 points	T1, T2, T3
Decrease in cardiac anxiety by 0.2 points	Total score of the German 17-item version of the CAQ [16]	Binary, yes/no	T1, T2, T3
Depression	PHQ-D [25]	Continuously, 0 to 27 points	T0, T1, T2, T3
Generalized anxiety	PHQ-D [25]	Continuously, 0 to 21 points	T0, T1, T2, T3
Somatization	PHQ-D [25]	Continuously, 0 to 30 points	T0, T1, T2, T3
Functional capacity	IRES-24 [26]	Continuously, 0 to 10 points	T0, T1, T2, T3
Blood pressure	Clinical examination	Continuously, systolic/diastolic in mmHg	T0, T1
Endurance	Clinical examination	Continuously, ergometer performance in watts/kg	T0, T1
General health	Visual analog scale of the EQ-5D [27]	Continuously, 0 to 100 points	T0, T1, T2, T3
Health-related quality of life	EQ-5D [28]	Continuously, health state of 11111 corresponds to a value of 1	T0, T1, T2, T3
Motivation to change lifestyle	OutCaRe [31]	Binary, certainly or rather yes vs. uncertain or less	T0, T1
Weekly physical activity	BSA-F [33]	Continuously, minutes	T0, T1, T2, T3
Self-efficacy to exercise	ESES [32]	Continuously, 10 to 40 points	T0, T1, T2, T3
Smoking status	Own development	Binary, yes/no	T0, T1, T2, T3
Weight	Clinical examination	Continuously, body mass index in kg/m²	T0, T1, T2, T3
Self-rated work ability	Work Ability Score [34]	Continuously, 0 to 10 points	T0, T1, T2, T3
Sickness absence	Own development	Binary, yes/no	T0, T2, T3
Sickness absence duration	Own development	Continuously, cumulative sickness absence in the last 3 or 12 months in weeks	T0, T2, T3
Stable return to work	[36]	Binary, yes/no	T2, T3
Time to stable return to work	[36]	Continuously, weeks	T2, T3
Employment	Own development	Binary, yes/no	T0, T2, T3
Disability pension	Own development	Binary, yes/no	T0, T2, T3
Capacity for last job	Medical assessment, standardized medical discharge report [37]	Binary, at least six hours per day or less than six hours per day	T1
Capacity for other job	Medical assessment, standardized medical discharge report [37]	Binary, at least six hours per day or less than six hours per day	T1
Treatments during the rehabilitation program	Standardized medical discharge report [37], coding according to the classification of therapeutic services report [39]	Continuously, minutes or hours	T1
Content of the cognitive-behavioral rehabilitation program	[14]	Continuously, 0 to 12 points	T1
Consistency of the approach	[14]	Continuously, 0 to 16 points	T1
Gain in competence	[14]	Continuously, 0 to 40 points	T1
Recommendations for subsequent services	Standardized medical discharge report [37]	Binary, yes/no for 12 subsequent services	T1
Utilization of medical and non-medical services	FIMA [40]	Continuously, resource usage in euro	T3
Diagnoses	Standardized medical discharge report [37], clinical interview[42]	Nominal	T1
Sociodemographic data	Own development	Various (native language, number of children, level of school education, vocational qualification)	T0

*BSA-F* Physical Activity, Exercise, and Sport Questionnaire (German: Bewegungs- und Sportaktivität Fragebogen), *CAQ* Cardiac Anxiety Questionnaire, *ESES* Exercise Self-Efficacy Scale, *EQ-5D* European Quality of Life 5 Dimensions, *FIMA* Questionnaire for Health-Related Resource Use in an Elderly Population (German: Fragebogen zur Erhebung von Gesundheitsleistungen im Alter), *IRES-24* Indicators of rehabilitation status (German: Indikatoren des Reha-Status), *OutCaRe* Outcome of Cardiac Rehabilitation, *PHQ-D* Patient Health Questionnaire, *T0* Beginning of the rehabilitation, *T1* End of the rehabilitation, *T2* 3-month follow-up, *T3* 12-month follow-up

### Harms

Although adverse events causally related to cognitive-behavioral rehabilitation are not expected, we will assess disability pensions and premature termination of rehabilitation in both groups.

### Ancillary and post-trial care

Ancillary and post-trial care are not planned. Compensation for harms due to study participation is also not planned.

### Participant timeline

Table 3 shows the full schedule of recruitment, interventions, and assessments.

**Table 3:** Schedule of enrollment, intervention, and assessments

*Activity*	*Responsibility*	*Duration*	*P*	*T0*	*T1*	*T2*	*T3*
**Recruitment**							
Written information	Rehabilitation center	10 min	x				
Information by telephone	Rehabilitation center	10 min	x				
Randomization	Rehabilitation center, principal investigator	5 min	x				
**Intervention**							
Rehabilitation	Rehabilitation center	4 weeks		x	x		
**Assessments**							
Clinical examination	Rehabilitation center	45 min		x	x		
Documentation of treatment	Rehabilitation center	15 min			x		
Questionnaire	Principal investigator	30 min		x	x	x	x

*P* Preparation, *T0* Beginning of the rehabilitation, *T1* End of the rehabilitation, *T2* 3-month follow-up, *T3* 12-month follow-up

### Sample size

As a minimum important difference for the 12-month follow-up, we defined 0.2 points of the total cardiac anxiety score (0 to 4 points [15, 16]). Although 0.2 points represent only 5% of the scale range, we consider this small gain to be relevant because we are comparing two comprehensive rehabilitation programs. Assuming a standard deviation of 0.6 points [43], 0.2 points correspond to a standardized mean difference of 0.33. To detect this difference with a two-sided error of 5% and a power of 80%, 286 participants are required. Further assuming a response rate of 70% in the 12-month follow-up, we plan to recruit 410 participants (intervention group: n = 205; control group: n = 205) to be able to detect the minimal important difference also in a complete-case analysis. For our primary intention-to-treat analysis with multiple imputation of missing follow-up scores, a difference of 0.2 points is detected with a power of about 92%.

### Recruitment

Study participants are recruited at a German inpatient rehabilitation center (https://www.muehlenbergklinik-holsteinische-schweiz.de). Eligibility is determined prior to the start of rehabilitation either by the referring pension insurance agency or by the rehabilitation center if a mild or moderate mental illness or distress or exhaustion is described in the application documents. A physician from the study center therefore continuously screens the incoming application documents of patients who are assigned to the rehabilitation center by the pension insurance agencies. A member of the study team (psychologist) in the study center informs potential participants in writing about the possibility of participating in the study, specifies the possible start of rehabilitation, and suggests a telephone consultation to the participants. The relaying of this information by telephone is supported by a manual. After receiving verbal information during the telephone conversation, the potential participants sign the consent form and return it to the rehabilitation center within 48 h. The written information and the consent form are provided as Additional files 3 and 4. A website provides supplementary and ongoing information about the study (https://www.vor-kardio.de).

### Allocation

Randomization is performed in a one-to-one ratio. The randomization sequence was generated by the principal investigator (MB) using Stata 16.0. Blocks of different size were combined randomly. Allocation concealment is ensured by sealed, opaque, and consecutively numbered envelopes. The envelopes are opened in the study center for randomization only after participants have given informed consent. The group assignment is documented in the study center in the study list through the identification number.

### Blinding

Participants and providers are not blinded. The data analysis is also not blinded.

### Data collection methods

Data are collected via questionnaires, clinical examinations, and the standardized rehabilitation discharge reports. Questionnaires are completed at four measurement points: at the beginning and end of rehabilitation, as well as 3 and 12 months after completion of rehabilitation. The assessments have already been tested during the implementation of the intervention [14] and are described in detail above (Table 2). The 3-month and 12-month follow-up questionnaires are sent from the rehabilitation center. Patients whose follow-up questionnaires have not been received after two weeks are reminded twice at two-week intervals, if necessary. We enclose the questionnaire again with each reminder. Clinical examinations are performed at the beginning and end of rehabilitation. Treatments administered during rehabilitation are documented by the rehabilitation center in the discharge reports at the end of rehabilitation.

### Data management

Data from questionnaires are entered by trained research assistants. We use forms created with Microsoft Access for data entry. When entering the data, the research assistants can only select from a set of valid values. The first 20 questionnaires of each of the four measurement points and subsequently every 20th questionnaire are re-entered by a second independent person. Incorrect completion of questionnaires (e.g., multiple crosses) are logged. Data entries are made immediately when the questionnaires are available to the University of Lübeck. The presence of the questionnaires at the beginning and end of the rehabilitation is documented in the study list of the rehabilitation center. The same applies to the mailing of the 3-month and 12-month follow-up questionnaires. The questionnaires received after 3 and 12 months are documented at the University of Lübeck. The identification numbers of the incoming questionnaires are transmitted by the University of Lübeck to the rehabilitation center. If receipt is confirmed by the University of Lübeck, no reminder is sent. A comprehensive plausibility check is performed for all data prior to our analysis of the data.

### Data monitoring

An external data monitoring committee is not implemented. Short-term and long-term effects are analyzed and published separately. No further interim analyses are planned. No criteria for early study termination have been established.

### Auditing

The status of recruitment, the treatment fidelity, the response rates of the questionnaires, and the number of questionnaires entered are discussed in bi-weekly video conferences between the Universität zu Lübeck and the rehabilitation center.

### Confidentiality

Participants are added to a password-protected study list (Microsoft Excel) with their name, address, and social security number upon consent to participate and are assigned an identification number. This list also documents the treatment group, consent, and the planned start of rehabilitation after randomization. This study list remains at the recruiting rehabilitation center. The rehabilitation center transmits a pseudonymized copy of this list exclusively with the pseudonym, treatment group, date of receipt of consent and planned start of rehabilitation electronically and encrypted to the University of Lübeck at the beginning of each week. The study list will be shredded by the rehabilitation center after all follow-up questionnaires have been sent out. As of that point, the data will be completely anonymized.

The rehabilitation center documents in another list (Microsoft Excel), in which participants are recognized only by their identification number (pseudonymized list), their date of birth, gender, the actual start of rehabilitation and the end of rehabilitation (if necessary, also the early termination of rehabilitation), selected clinical data collected in the rehabilitation center (systolic and diastolic blood pressure, ergometer performance in watts/kg, capacity for last job and other job, recommendations for subsequent services, diagnoses), and the therapeutic services performed during rehabilitation.

The participants’ questionnaires are provided with the identification number in the rehabilitation center and handed over to the study participants by a member of the research team for completion at the beginning and end of the rehabilitation. The participants deliver the questionnaires in an envelope at the reception of the rehabilitation center. Thereafter, these questionnaires are personally handed over to MB by DB every two weeks. The questionnaires of the follow-up assessments after 3 and 12 months, also tagged with the identification number, as well as the reminders, are sent from the rehabilitation center. A stamped return envelope addressed to the University of Lübeck is enclosed with this letter.

Data, pseudonymized with the identification number, are recorded electronically at the University of Lübeck using Microsoft Access. All questionnaires received by the University of Lübeck are stored in locked cabinets at the University of Lübeck and will be shredded in accordance with data protection regulations upon completion of the study on 28.02.2025. Computers at the University of Lübeck that allow access to the data entered are located in locked office rooms and require user authentication in the form of passwords. Accesses can be tracked. Data is stored in folders that are only visible and accessible to the research team at the University of Lübeck. The network in which the computers are integrated is protected against external access and manipulation by a regularly updated firewall system. The University of Lübeck will completely delete the electronically stored questionnaire data 10 years after the end of the study (28.02.2035). To ensure a transparent research process, anonymized data will be permanently stored in a data repository after our primary publication is accepted (https://www.synapse.org).

### Access to data

All authors of the study protocol will have access to the final and fully anonymized data set.

### Statistical methods

We will analyze short-term (end of rehabilitation and 3-month follow-up) and long-term effects (12-month follow-up) using different models and publish the findings separately. We will use mixed models if more than one measurement point needs to be considered, i.e., end of rehabilitation and 3-month follow-up, and generalized linear models otherwise. If baseline scores are available, these will be included as a covariate when estimating the treatment effect. If outcomes are binary, a logit link will be used. High depression, high generalized anxiety, and high cardiac anxiety, as well as age, sex, and education, are tested as possible moderators of the treatment effect on our primary outcome cardiac anxiety assessed at the 12-month follow-up.

For health economic evaluation, we will calculate an incremental cost-effectiveness ratio [44]. Assessments of costs employ a societal perspective [41, 45]. Effectiveness is measured using quality-adjusted life years as derived from the 12-month assessment of the EQ-5D-5 L [28, 30]. The incremental cost-effectiveness ratio is calculated by dividing the difference in costs between both interventions by the difference in effectiveness between both interventions. Bootstrapping is used to compute a 95% confidence interval of the incremental cost-effectiveness [44].

Missing data will be imputed using chained equations [46]. We will create 20 independent data sets with complete values. Parameter estimates of the models will be combined in accordance with Rubin’s rules [47]. Statistical tests will be regarded as significant if the two-sided p-value of a test is less than 0.05. An up-to-date version of Stata (StataCorp, College Station, Texas, USA) will be used to conduct the analyses.

## Discussion

The purpose of our randomized controlled trial is to test the effects of a cognitive-behavioral rehabilitation program for individuals with cardiovascular disease and mild or moderate mental illness or stress or exhaustion, in comparison with standard cardiac rehabilitation. Our study will provide high-quality evidence to inform pension insurance providers if cognitive-behavioral rehabilitation is likely to improve outcomes in similar patients and could be recommended for implementation across Germany. All results of our study will be published as articles in peer-reviewed journals and at conferences, regardless of the magnitude or direction of the effects. The authors of this protocol will write the final trial publications. We do not intend to use professional writers. We will provide updated information on the trial on our website https://www.vor-kardio.de. This study protocol was prepared using the SPIRIT checklist (Standard Protocol Items: Recommendations for Interventional Trials) [48].

## Trial status

Recruitment has started and is ongoing.

## Electronic supplementary material

Below is the link to the electronic supplementary material.

Additional file 1: Items from the World Health Organization Trial Registration Data Set.

Additional file 2: Description of the components of the cognitive-behavioral rehabilitation program according to the TIDieR checklist.

Additional file 3: Patient information.

Additional file 4: Consent to study participation.

## Data Availability

The datasets used and/or analyzed during the current study are available from the corresponding author on reasonable request.

## Abbreviations

BSA-F: Physical Activity, Exercise, and Sport Questionnaire (German:Bewegungs- und Sportaktivität Fragebogen)
CAQ: Cardiac Anxiety Questionnaire
ESES: Exercise Self-Efficacy Scale
EQ-5D: European Quality of Life 5 Dimensions
FIMA: Questionnaire for Health-Related Resource Use in an Elderly Population (German:Fragebogen zur Erhebung von Gesundheitsleistungen im Alter)
IRES-24: Indicators of rehabilitation status (German:Indikatoren des Reha-Status)
OutCaRe: Outcome of Cardiac Rehabilitation
PHQ-D: Patient Health Questionnaire
SPIRIT: Standard Protocol Items:Recommendations for Interventional Trials
T0: Beginning of the rehabilitation
T1: End of the rehabilitation
T2: 3-month follow-up
T3: 12-month follow-up
