# Supplementary material for: Cognitive-behavioral rehabilitation in patients with cardiovascular diseases: a randomized controlled trial (CBR-CARDIO, DRKS00029295)
Source: BMC Cardiovasc Disord. 2023 May 15;23:252. doi: 10.1186/s12872-023-03272-1 (PMC10186766; doi:10.1186/s12872-023-03272-1)
Supplement: Supplementary file 1 — Additional file 1: Items from the World Health Organization Trial Registration Data Set. [file 12872_2023_3272_MOESM1_ESM.docx]

# Additional file 1: Items from the World Health Organization Trial Registration Data Set

| **Data category** | **Information** |
| --- | --- |
| Register | German Clinical Trials Register |
| Last refreshed on | 4 October 2022 |
| Main ID | DRKS00029295 |
| Date of registration | 21/06/2022 |
| Prospective registration | Yes |
| Primary sponsor | Universität zu Lübeck, Institut für Sozialmedizin und Epidemiologie |
| Public title | Cognitive-behavioral rehabilitation in patients with cardiovascular diseases: A randomized controlled trial |
| Scientific title | Cognitive-behavioral rehabilitation in patients with cardiovascular diseases: A randomized controlled trial – CBR-CARDIO |
| Date of first enrolment | 22/06/2022 |
| Target sample size | 410 |
| Recruitment status | Pending |
| URL | http://www.drks.de/DRKS00029295 |
| Study type | Interventional |
| Allocation | Randomized controlled trial |
| Masking | Open (masking not used) |
| Control | Active control (effective treatment of control group) |
| Assignment | Parallel |
| Purpose | Treatment |
| Phase | N/A |
| Countries of recruitment | Germany |
| Contacts | Matthias Bethge Ratzeburger Allee 160, 23562 Lübeck, Germany +49 451 50051280 [matthias.bethge@uksh.de](mailto:matthias.bethge@uksh.de) Universität zu Lübeck, Institut für Sozialmedizin und Epidemiologie |
| Key inclusion and exclusion criteria | Inclusion criteria: We will include patients aged 18 to 65 years who are receiving rehabilitation at our study center due to a cardiovascular disease (ICD-10 I05 to I71 as well as I95 and I97), assigned by the Federal German Pension Insurance or the German Pension Insurance North, and for whom mild or moderate mental illness or stress or exhaustion are recorded in the application documents for medical rehabilitation. For patients assigned by the Federal German Pension Insurance, mental illness or stress or exhaustion is determined by the socio-medical service of the Federal German Pension Insurance. For patients assigned by the German Pension Insurance North, this is determined by a cardiologist from the study center. The determination of mild or moderate mental illness, stress, or exhaustion will be done in advance of the program with only the documents used to claim rehabilitation.  Exclusion criteria: Patients with severe mental illness (schizophrenia, schizoaffective disorder, bipolar disorder, mania, severe unipolar depression), severe heart failure (at least NYHA stage III), and significant limitations with the German language will be excluded.  Age minimum: 18 years  Age maximum: 65 years  Gender: Both male and female |

| Health conditions or problems studied | Cardiovascular disease (I05 to I71, as well as I95 or I97, ICD-10) and mild or moderate mental illness or stress or exhaustion  I05-I09 - Chronic rheumatic heart diseases  I10-I15 - Hypertensive diseases  I20-I25 - Ischemic heart diseases  I26-I28 - Pulmonary heart disease and diseases of the pulmonary circulation  I30-I52 - Other forms of heart disease  I60-I69 - Cerebrovascular diseases  I70 - Atherosclerosis  I71 - Aortic aneurysm and dissection  I95 - Hypotension  I97 - Postprocedural disorders of circulatory system not otherwise classified |
| --- | --- |
| Interventions | Intervention 1: Four-week cognitive-behavioral cardiac rehabilitation program. The program complements conventional cardiac rehabilitation with additional psychological interventions and exercise therapy and follows the framework of cognitive-behavioral rehabilitation, which was formulated by the German pension insurance fund (Deutsche Rentenversicherung, 2016). A description of the program is provided in Benninghoven et al. (2022; <https://doi.org/10.1055/a-1749-6379>).  Intervention 2: Four-week cardiac rehabilitation program in accordance with the guideline for the rehabilitation of cardiovascular disease (Deutsche Rentenversicherung, 2020). |
| Primary outcome | Cardiac anxiety. How? Continuously, 0 to 4 points, total score of the German 17-item version of the Cardiac Anxiety Questionnaire (Hoyer et al., 2008). When? Start and end of the rehabilitation, 3-month follow-up, and 12-month follow-up. Our primary outcome is cardiac anxiety 12 months after the end of rehabilitation. |
| Secondary outcomes | Fear. How? Continuously, 0 to 4 points, subscale of the German 17-item version of the Cardiac Anxiety Questionnaire (Hoyer et al., 2008). When? Start and end of the rehabilitation, 3-month follow-up, and 12-month follow-up.  Avoidance. How? Continuously, 0 to 4 points, subscale of the German 17-item version of the Cardiac Anxiety Questionnaire (Hoyer et al., 2008). When? Start and end of the rehabilitation, 3-month follow-up, and 12-month follow-up.  Attention. How? Continuously, 0 to 4 points, subscale of the German 17-item version of the Cardiac Anxiety Questionnaire (Hoyer et al., 2008). When? Start and end of the rehabilitation, 3-month follow-up, and 12-month follow-up.  Change score of cardiac anxiety. How? Continuously, −4 to 4 points, total score of the German 17-item version of the Cardiac Anxiety Questionnaire (Hoyer et al., 2008). When? End of the rehabilitation, 3-month follow-up, and 12-month follow-up.  Decrease in cardiac anxiety by 0.2 points. How? Yes/no, total score of the German 17-item version of the Cardiac Anxiety Questionnaire (Hoyer et al., 2008). When? End of the rehabilitation, 3-month follow-up, and 12-month follow-up.  Depression. How? Continuously, 0 to 27 points, PHQ-D (Kroenke et al., 2010). When? Start and end of the rehabilitation, 3-month follow-up, and 12-month follow-up.  Generalized anxiety. How? Continuously, 0 to 21 points, PHQ-D (Kroenke et al., 2010). When? Start and end of the rehabilitation, 3-month follow-up, and 12-month follow-up.  Somatization. How? Continuously, 0 to 30 points, PHQ-D (Kroenke et al., 2010). When? Start and end of the rehabilitation, 3-month follow-up, and 12-month follow-up.  Functional capacity. How? Continuously, 0 to 10 points, IRES-24 (Wirtz et al., 2005). When? Start and end of the rehabilitation, 3-month follow-up, and 12-month follow-up.  Blood pressure. How? Continuously, systolic/diastolic in mmHg. When? Start and end of the rehabilitation.  Endurance. How? Continuously, ergometer performance in watts/kg. When? Start and end of rehabilitation.  General health. How? Continuously, 0 to 100 points, visual analog scale of the EQ-5D (Radin and de Charro, 2001). When? Start and end of the rehabilitation, 3-month follow-up, and 12-month follow-up.  Health-related quality of life. How? Continuously, a health state of 11111 corresponds to a value of 1, EQ-5D (Herdman et al., 2011). When? Start and end of the rehabilitation, 3-month follow-up, and 12-month follow-up.  Motivation to change lifestyle. How? Proportion certainly or rather yes (Zoch-Lesniak et al., 2020). When? Start and end of the rehabilitation.  Weekly physical activity. How? Continuously, minutes, BSA-F (Adams et al., 2021). When? Start and end of the rehabilitation, 3-month follow-up, and 12-month follow-up.  Self-efficacy to exercise. How? Continuously, 10 to 40 points, Exercise Self-Efficacy Scale (Kroll et al., 2007). When? Start and end of the rehabilitation, 3-month follow-up, and 12-month follow-up.  Smoking status. How? Yes/no. When? Start and end of the rehabilitation, 3-month follow-up, and 12-month follow-up.  Weight. How? Continuously, body mass index in kg/m². When? Start and end of the rehabilitation, 3-month follow-up, and 12-month follow-up.  Self-rated work ability. How? Continuously, 0 to 10 points, Work Ability Score (Ilmarinen, 2007). When? Start and end of the rehabilitation, 3-month follow-up, and 12-month follow-up.  Sickness absence. How? Yes/no. When? Start of the rehabilitation, 3-month follow-up, and 12-month follow-up.  Sickness absence duration. How? Cumulative sickness absence the past 3 or 12 months in weeks. When? Start of the rehabilitation, 3-month follow-up, and 12-month follow-up.  Return to work. How? Stable return to work (yes/no) and time to stable return to work in weeks (continuously) (Kuijer et al., 2012). When? 3-month follow-up, and 12-month follow-up.  Employment. How? Yes/no. When? Start of the rehabilitation, 3-month follow-up, and 12-month follow-up.  Disability pension. How? Yes/no. When? Start of the rehabilitation, 3-month follow-up, and 12-month follow-up.  Capacity for last job. How? At least six hours per day or less than six hours per day, medical assessment, standardized medical discharge report (Deutsche Rentenversicherung, 2014). When? End of the rehabilitation.  Capacity for other jobs. How? At least six hours per day or less than six hours per day, medical assessment, standardized medical discharge report (Deutsche Rentenversicherung, 2014). When? End of the rehabilitation.  Treatments during the rehabilitation program. How? Coding according to the classification of therapeutic services report (Deutsche Rentenversicherung, 2015), standardized medical discharge report (Deutsche Rentenversicherung, 2014). When? End of the rehabilitation.  Content of the cognitive-behavioral rehabilitation program. How? Continuously, 0 to 12 points (Benninghoven et al., 2022). When? End of the rehabilitation.  Consistency of the approach. How? Continuously, 0 to 16 points (Benninghoven et al., 2022). When? End of the rehabilitation.  Gain in competence. How? Continuously, 0 to 40 points (Benninghoven et al., 2022). When? End of the rehabilitation.  Recommendations for subsequent services. How? Yes/no for twelve subsequent services, standardized medical discharge report (Deutsche Rentenversicherung, 2014). When? End of the rehabilitation.  Utilization of medical and non-medical services. How? Continuously, resource usage in euros, Questionnaire for Health-Related Resource Use in an Elderly Population (FIMA) (Seidl et al., 2015). When? 12-month follow-up.  Sociodemographic data. How? Native language, number of children, level of school education, and vocational qualification. When? Start of the rehabilitation. |
| Secondary ID(s) | 22-160 (Ethics Committee University of Lübeck)  U1111-1264-6426 (Universal Trial Number) |
| Source of monetary support | Federal German Pension Insurance (Deutsche Rentenversicherung Bund) |
| Status of ethics review | Approved |
| Approval date of ethics review | 11/05/2022 |
