# Supplementary material for: Cognitive-behavioral rehabilitation in patients with cardiovascular diseases: a randomized controlled trial (CBR-CARDIO, DRKS00029295)
Source: BMC Cardiovasc Disord. 2023 May 15;23:252. doi: 10.1186/s12872-023-03272-1 (PMC10186766; doi:10.1186/s12872-023-03272-1)
Supplement: Supplementary file 2 — Additional file 2: Description of the components of the cognitive-behavioral rehabilitation program according to the TIDieR checklist. [file 12872_2023_3272_MOESM2_ESM.docx]

# Additional file 2: Description of the components of the cognitive-behavioral rehabilitation program according to the TIDieR checklist

| Brief name | Psychological group | Individual psychological sessions | Relaxation training | Seminar: Heart and psyche | Exercise group |
| --- | --- | --- | --- | --- | --- |
| Why | Thoughts, attitudes, and feelings influence the experience of illness and the way a person deals with it. In the long term, they also influence the prognosis of cardiovascular diseases. The psychological group intervention following acceptance and commitment therapy attempts to promote a way of dealing with aversive thoughts and emotions that enables flexible, value-driven behavior. Techniques are taught that enable individuals to view their own thoughts without merging with them (cognitive defusion) and necessarily aligning behavior with them.  The implementation in the group enables reciprocal social learning. The patients are given the opportunity to exchange experiences and to reflect together. Social comparison is made possible and mutual social support is encouraged. | During the admission interview, the therapy goals will be clarified. If necessary, additional individual meetings will supplement the therapeutic process of the group intervention. | Progressive muscle relaxation will be learned in order to use it specifically for pain or stress management. Progressive muscle relaxation can reduce physical restlessness or agitation. The use of progressive muscle relaxation lowers muscle tension below the previously normal level due to improved body awareness. Participants learn to induce muscular relaxation whenever they wish. | Being well informed is an important prerequisite for meaningful and self-determined behavioral changes. In the seminar, knowledge will be provided about the relationship between the functioning of the cardiovascular system and psychological well-being. | In the exercise group, the rehabilitation patients are taught that physical activity is possible despite their functional limitations and that it improves functioning and promotes vitality. Self-management skills and the transfer to everyday life are supported by educational content, and participants improve their physical functions (including coordination and strength). |
| What (materials) | Moderator's case, metaplan boards, flipchart, beamer and laptop, fact sheets and worksheets | Pens, flipchart | Mats and pads or comfortable chairs | Moderator's case, metaplan boards, flipchart, beamer, and laptop | Strength training equipment, mats, chairs, and pads |
| What (procedures) | The following topics are covered in the seven sessions.  Session 1: Introducing the program and getting to know each other; introducing the biopsychosocial treatment concept using the interaction between cardiovascular disease and the psychological state as an example; working out rehabilitation goals and group rules.  Session 2: Health-promoting behavior; cardiovascular disease and emotions; introducing and teaching the basic principles of mindfulness exercise; performing mindfulness exercise; psychoeducation: health-promoting behavior in cardiovascular disease; anxiety in cardiovascular disease; interaction of body and mind; emotions and the influence of stress on cardiovascular disease.  Session 3: Introduction of the ACT matrix and practice of group exercises; acceptance of negative thoughts; feelings and body sensations as a focus for change.  Session 4: Mental flexibility; dealing with contextually dysfunctional cognitions: introduction of the concept of cognitive fusion/defusion; group exercise on defusion; further application of the ACT matrix using concrete personal situations; further acceptance orientation.  Session 5: Identification of personal values (group exercise); acting on values as an alternative to unsuccessful control of emotional experience; derivation of concrete behaviors.  Session 6: Introduction of the concept of acceptance; exposure exercises.  Session 7: Evaluation of treatment: what helped? Elaboration of concrete plans for the time after rehabilitation (commitments); elaboration of goals (specific, measurable, attractive, realistic, time-bound) considering personal values. | During the admission interview, anamnestic information (including contextual factors such as family, work, and biography) as well as the experience of illness and illness behavior are recorded, and the expectations of the rehabilitation patients are clarified.  In addition, at the end of rehabilitation, mental disorders are assessed with a standardized short interview in order to specify further treatment recommendations. | Participants learn progressive muscle relaxation and develop individual ways to apply the technique in everyday life. | The following topics will be covered in the two sessions.  Session 1: Structure and function of the heart and cardiovascular system; function of the autonomic nervous system and the hormonal system.  Session 2: Control function of the brain and influence of the emotional state; influence of lifestyle and attitude changes on physical processes, especially on the cardiovascular system. | The exercise intervention includes an individual admission assessment by a physical therapist, cardiovascular exercise therapy, ergometer training, strength training, and coordination training in a group. |
| Who provided | A psychological psychotherapist or graduated psychologist in psychotherapy training leads the group throughout the rehabilitation and is the primary psychological contact. The primary physical therapist is present at the first and last group meetings. | Psychological psychotherapist or graduated psychologist in psychotherapy training | Psychological psychotherapist or graduated psychologist in psychotherapy training | Physician | The primary physiotherapist will lead the group exercise sessions throughout rehabilitation. |
| How | Group intervention | Individual intervention | Group intervention | Group intervention | Group intervention |
| Where | Quiet group room | Workroom of the primary psychologist | Quiet group room/gym | Quiet group room in the rehabilitation facility | Gym |
| When and how much | During the rehabilitation program, seven sessions of 90 minutes each, in total 630 minutes | During the rehabilitation program, at least one 60-minute session | During the rehabilitation program, one session of 60 minutes and eight sessions of 30 minutes each, in total 300 minutes | During the rehabilitation program, two sessions of 60 minutes each, in total 120 minutes | During the rehabilitation program, individual admission assessment: one sessions of 30 minutes, cardiovascular exercise therapy: 20 sessions of 30 minutes each, ergometer training: one session of 60 minutes and 11 sessions of 30 minutes each, strength training: 6 sessions of 60 minutes each, coordination training: 10 sessions of 30 minutes each, in total 1680 minutes |
| Tailoring | Specific topics that arise in the group are included. | Content is based on the individual needs of the patient. | Not planned. | Not planned. | Content of cardiovascular exercise therapy, strength training, and coordination training will be based on patients’ individual needs. Ergometer training will be standardized. |
| How well | All treatment components will be documented in the standardized rehabilitation discharge letters to assess the actual delivered dose. The rehabilitation teams will use the corresponding codes of the classification of therapeutic interventions developed by the German Pension Insurance for quality assurance in rehabilitation. In addition, patients will be asked about content and achievement of therapy goals at the end of the rehabilitation program with a standardized set of questions.  Weekly interdisciplinary meetings are conducted to discuss patients and the program implementation.  Moreover, we support fidelity of implementation of the psychological group regularly by participatory observation of selected group therapy sessions and a standardized adherence rating of these sessions, and the psychologists will meet every four weeks to discuss and harmonize continuously the content of the group therapy sessions. Standardized implementation of the psychological group will be ensured by common working materials (e.g., slides and fact sheets). | | | | |
