# Supplementary material for: Cognitive-behavioral rehabilitation in patients with cardiovascular diseases: a randomized controlled trial (CBR-CARDIO, DRKS00029295)
Source: BMC Cardiovasc Disord. 2023 May 15;23:252. doi: 10.1186/s12872-023-03272-1 (PMC10186766; doi:10.1186/s12872-023-03272-1)
Supplement: Supplementary file 3 — Additional file 3: Patient information. [file 12872_2023_3272_MOESM3_ESM.docx]

**Patient information**

**Cognitive-behavioral rehabilitation in patients with cardiovascular diseases: A randomized controlled trial**

Dear Ms. ..., Dear Mr. ...,

We kindly invite you to participate in our study on the effectiveness of a new cardiac rehabilitation program. The research project is funded by the Federal German Pension Insurance and is being carried out under the supervision of Prof. Dr. Matthias Bethge from the Institute for Social Medicine and Epidemiology at the University of Lübeck and PD Dr. Dieter Benninghoven from the Mühlenbergklinik.

**What issues do we want to clarify?**

Our study is intended to clarify whether the cognitive-behavioral rehabilitation offered at the rehabilitation center can reduce participants' cardiac anxiety to a greater extent than the standard cardiac rehabilitation currently offered. For this purpose, we have supplemented the rehabilitation with, e.g., a psychological group that supports mindfulness.

**What is cognitive-behavioral rehabilitation?**

Cognitive-behavioral rehabilitation is a treatment for patients who experience mild or moderate mental illness, stress, or exhaustion in addition to the primary cardiovascular disease that led to rehabilitation. Cognitive-behavioral rehabilitation programs have already proven beneficial in musculoskeletal and cancer rehabilitation. With our study, we want to test the effects of such a program for patients with cardiovascular disease.

**What is the difference between cognitive-behavioral and standard cardiac rehabilitation?**

The cognitive-behavioral program complements standard rehabilitation with additional psychological and exercise interventions. A characteristic feature of cognitive-behavioral rehabilitation is the closed group. Eight to twelve patients are treated together in specific cognitive-behavioral psychological and exercise interventions.

**How will the study proceed?**

In our study, the participants will be assigned randomly to either cognitive-behavioral rehabilitation or standard cardiac rehabilitation. This is the only way we can test whether the complementary offering has an additional effect. To ensure that any potential benefits of the cognitive-behavioral rehabilitation program are not due to the longer rehabilitation duration of the cognitive-behavioral program, the standard rehabilitation program will also be conducted for four weeks instead of the usual three weeks.

**General information on data protection**

In accordance with the European Data Protection Regulation (EU-DSGVO), we would like to inform you about the data we will collect during the study and the purpose of the data we will collect. In addition, we will inform you about your rights.

**Which data will be collected?**

Two different data sources will be used for the study. First, we will assess questionnaire data at the beginning and end of rehabilitation and after 3 and 12 months. The questionnaires cover topics such as your state of health, the use of other health services, and the content of your rehabilitation. The questionnaires take about 30 minutes to complete. You will receive the first two questionnaires at the Mühlenbergklinik. The other two will be sent to your home by the Mühlenbergklinik. You will then return the completed questionnaires to the University of Lübeck in a prepaid envelope. Secondly, data collected at the Mühlenbergklinik from physical examinations (blood pressure, weight, height, ergometer performance) as well as data from your medical discharge report (treatments during rehabilitation, capacity for last job or another job, recommendations for subsequent services, discharge diagnoses) are submitted to the University of Lübeck using an identification number. For this purpose, the staff of the rehabilitation center are released from their duty of confidentiality.

**How do we handle the collected data?**

If you have given your consent to participate in our study, your name and address will be added to a study list at the Mühlenbergklinik, in which you will be assigned an identification number. This list will remain at the Mühlenbergklinik and will be deleted on 28.02.2025. The questionnaires you receive will be marked with your identification number. All data described above will finally be linked at the University of Lübeck under the identification number and evaluated by Prof. Dr. Matthias Bethge. The analyses are pseudonymized, i.e., they only include your identification number, but no names or other personal details. In order to ensure a transparent research process, the completely anonymized data will be permanently stored in a data repository (https://www.synapse.org/).

**Voluntariness of participation and your rights**

Participation in the study is voluntary. If you decide not to participate, you will not suffer any disadvantages. You will then receive the conventional three-week cardiac rehabilitation. Participation in the study is not associated with any risks: the program being tested in our study has been evaluated in an implementation study. Adverse events causally related to the intervention being tested are not expected. If you wish to withdraw your participation at a later date, obtain information about your stored data, assert restriction of the processing of your data, or use your right to data deletion, please contact Prof. Dr. Matthias Bethge at the University of Lübeck (e-mail: matthias.bethge@uksh.de). The withdrawal will result in the irreversible deletion of the collected data. Deletion of the data after withdrawal is only possible until the time of complete anonymization of the data. Participation in cognitive-behavioral rehabilitation without participation in this scientific study is not possible.

In case of a complaint, please contact:

Independent State Center for Data Protection Schleswig-Holstein

Holstenstraße 98, 24103 Kiel

Email: [mail@datenschutzzentrum.de](mailto:mail@datenschutzzentrum.de)

**Responsibility for the research project and responsible data protection officer**

Prof. Dr. Matthias Bethge from the University of Lübeck is responsible for the research project (e-mail: matthias.bethge@uksh.de). If you have general questions about data protection, you can contact the data protection officer at the University of Lübeck: x-tention Informationstechnologie GmbH (Karl-Drais-Str. 4e, 86167 Augsburg, Germany, Tel.: +49 451 31011903, email: datenschutz@uni-luebeck.de).

**Request for participation**

If you would like to participate in the study, please call A. B. (phone: 0123 456) or C. D. (0123 789) at the Mühlenbergklinik. In any case, A. B. or C. D. will contact you within the next two weeks and inform you verbally about the study. You are also welcome to inform A. B. by email (A.B@studycenter.de) when you can best be reached by phone.

After the telephone conversation, you can – if you decide to participate – sign the consent form, which is available to you in duplicate, and send it to the Mühlenbergklinik. The other copy is intended for you. We would be very pleased to have you participate.

You will also find further information about our study on the website https://www.vor-kardio.de. There, we will inform you about our study.

Please do not hesitate to contact me if you have any questions!

We would like to thank you for your support and assistance and remain yours sincerely.

Signature of the principal investigator
