# Supplementary material for: Cognitive-behavioral rehabilitation in patients with cardiovascular diseases: a randomized controlled trial (CBR-CARDIO, DRKS00029295)
Source: BMC Cardiovasc Disord. 2023 May 15;23:252. doi: 10.1186/s12872-023-03272-1 (PMC10186766; doi:10.1186/s12872-023-03272-1)
Supplement: Supplementary file 4 — Additional file 4: Consent to study participation. [file 12872_2023_3272_MOESM4_ESM.docx]

**Consent to study participation**

**Cognitive-behavioral rehabilitation for patients with cardiovascular disease:
A randomized controlled trial**

…………………………………………… ……………………………………………

First name Last name

I was informed about the content and purpose of the study "Cognitive-behavioral rehabilitation for patients with cardiovascular disease: A randomized controlled trial." This study is funded by the Federal German Pension Insurance and is carried out under the responsibility of Prof. Dr. Matthias Bethge from the Institute for Social Medicine and Epidemiology at the University of Lübeck and PD Dr. Dieter Benninghoven from the Mühlenbergklinik.

I have received information on data protection issues and random assignment to one of the two rehabilitation programs (standard cardiac rehabilitation or cognitive-behavioral cardiac rehabilitation). I wish to support the study with my participation and agree to fill in the questionnaires that will be handed out to me or sent to me by the Mühlenbergklinik. I will not incur any costs as a result.

In addition, I agree that data from clinical examinations mentioned in the information (blood pressure, weight, height, ergometer performance, and diagnoses) may be used as part of the study, as well as data mentioned in the medical discharge report. I agree to the rehabilitation center forwarding these data to the University of Lübeck using a study number. In this respect, I release the staff of the rehabilitation center from their obligation to medical confidentiality.

I agree that the data from clinical examinations, the data from medical discharge reports, and the questionnaire data will be merged pseudonymously at the University of Lübeck under a study number. I have been assured that no personal data (name, date of birth, address) or other data that could be used to draw conclusions about my person will be passed on to third parties outside the study.

I am aware that I can withdraw my consent at any time without giving a reason and without disadvantage. I have been informed comprehensively about my rights and about the time expenditure incurred by me as a study participant. All my questions have been answered completely.

Under these conditions, I declare my consent to participate in the study and agree to the above-mentioned release from confidentiality.

………………………………………… …………………………………………

Place and date Signature
